# A paternal methyl donor depleted diet leads to increased anxiety- and depression-like behavior in adult rat offspring

**DOI:** 10.1042/BSR20180730

**Published:** 2018-07-06

**Authors:** Chelsea R. McCoy, Nateka L. Jackson, Rachel L. Brewer, Mohamad M. Moughnyeh, Daniel L. Smith, Sarah M. Clinton

**Affiliations:** 1 School of Neuroscience, Virginia Polytechnic Institute and State University, Blacksburg, VA, U.S.A.; 2 Department of Cellular, Developmental and Integrative Biology, University of Alabama at Birmingham, Birmingham, AL, U.S.A.; 3 Department of Nutrition Sciences, University of Alabama at Birmingham, Birmingham, AL, U.S.A.

**Keywords:** Anxiety, Depression, DNA Methylation, Diet, Epigenetics

## Abstract

Epigenetic mechanisms such as DNA methylation elicit lasting changes in gene expression and likely mediate gene–environment interactions that shape brain development, behavior, and emotional health. Myriad environmental factors influence DNA methylation, including methyl donor content in the paternal diet, could influence methylation in offspring via changes in the paternal germ line. The present study examines the effects of paternal methyl donor dietary deficiency on offspring’s emotional behaviors, including anxiety, social interaction, and depression-like behavior. We previously found that rats bred to display high levels of anxiety- and depression-like behavior exhibit diminished DNA methylation in the amygdala. We also observed that depleting dietary methyl donor content exacerbated the rats’ already high levels of anxiety- and depression-like behavior. Here we sought to determine whether paternal dietary methyl donor depletion elicits intergenerational effects on first generation (F1) offspring’s behavior (potentially triggering a similar increase in anxiety- and/or depression-like behavior). Thus, adult male rats prone to high anxiety/depression-like behavior, were fed either a methyl donor depleted (DEP) or control (CON) diet for 5 weeks prior to mating. They were paired with females and resultant F1 male offspring were subjected to a behavioral test battery in adulthood. F1-DEP offspring showed a similar behavioral profile to the F0 males, including greater depression-like behavior in the forced swim test (FST) and increased anxiety-like behavior in the open field test (OFT). Future work will interrogate molecular changes in the brains of F1 offspring that mediate these intergenerational effects of paternal methyl donor dietary content on offspring emotional behavior.

## Introduction

Epigenetic mechanisms lie at the crossroads where ‘nature’ meets ‘nurture’ and likely mediate gene–environment interactions that shape neurodevelopment, behavior, and neuropsychological health [1]. DNA methylation, one of the most studied epigenetic mechanisms, elicits enduring changes in gene expression by adding methyl groups to cytosine residues [2]. DNA methylation processes in the brain can be modified by myriad environmental factors [3–9], including diet, since dietary folate, choline, and methionine act as methyl donors for one-carbon transfer reactions like DNA methylation. DNA methyltransferase (DNMT) enzymes transfer methyl groups from *S*-adenosylmethionine (SAM) to cytosine [10], so diets lacking folate or other methyl donors can impede SAM synthesis, thereby leading to DNA hypomethylation [11–13]. Depleting dietary methyl donor content in adult animals decreases DNA methylation markers in the brain [13], impairs fear memory [14,15], and can induce depression-like behavior in susceptible individuals [16]. On the other hand, boosting levels of methyl donors (or SAM itself) increases DNA methylation levels in brain [17], and has been shown to improve anxiety- and depression-like behavior in rodents [16] and human clinical populations [18–20].

DNA methylation plays particularly critical roles in the developing organism since marked epigenetic programing and reprograming occurs throughout gestation and early postnatal life [21]. Consequently, early development represents a critical window when DNA methylation modifications can elicit lasting effects on brain and behavior. Females’ dietary methyl donor content during pregnancy influences various aspects of offspring development; for example maternal folate deficiency in rodents leads to decreased litter size, low birth weights, neural tube defects, and increased anxiety in adult offspring [22,23]. Fathers’ dietary methyl donor intake also influences offspring’s phenotype. One study found that paternal folate deficiency prior to mating leads to diminished DNA methylation in brains of offspring [24]; paternal methyl donor supplementation, on the other hand, leads offspring to display increased DNA methylation levels in the brain as well as altered hippocampal plasticity and memory capability [25]. The present study examines the effects of paternal methyl donor deficiency on emotional behaviors, including anxiety-like behavior, sociability, and helplessness (depression-like) behavior.

We have spent several years working with selectively bred lines of Sprague–Dawley rats that exhibit marked differences in emotional behavior and stress reactivity. Rats bred for low behavioral response to novelty (low responders, LRs) exhibit high levels of anxiety- and depression-like behavior compared with rats selected for high response to novelty (high responders, HRs) [26]. We have exploited the HR/LR model to examine neural circuit and molecular mechanisms that contribute to individual differences in emotional behavior. This work identified marked gene expression and epigenetic changes in multiple brain regions, particularly within the hippocampus and amygdala [16,27–30]. We observed DNA methylation changes in the early postnatal and adult HR/LR brain [16,30,31], including diminished DNA methylation levels in the adult LR compared with HR amygdala. We later found that increasing DNA methylation in LRs (via increased dietary methyl donor content) reduced their typically high levels of anxiety- and depression-like behavior, while decreasing methylation (via dietary methyl donor depletion) exacerbated LRs’ depression-like behavior [16]. The current study builds upon the earlier work to ask whether paternal dietary methyl donor depletion elicits intergenerational effects on LR offspring. Since dietary methyl donor depletion led to greater anxiety- and depression-like behavior in adult male LR rats, we hypothesized that such methyl donor depletion in LR F0 generation before mating would trigger heightened anxiety- and/or depression-like behavior in their offspring.

## Materials and methods

Experiments were approved by the local University Committee on the use and care of animals, and all work was performed in accordance with the National Institutes of Health (U.S.A., 2011) and National Research Council (U.S.A., 2011) guidelines on animal research.

### Animals

LR rats were obtained from the fourth generation of our in-house colony where the bred HR/LR lines have been maintained for several generations [16]. Housing and testing facilities were maintained at 21–23°C and 50–55% humidity. Rats were housed two to three per cage in a 12:12 light–dark cycle (lights on/off at 6 a.m./6 p.m.). Because our prior experiments found that manipulating dietary methyl donor content was effective in shifting adult LR rats’ behavioral phenotype [16], the present study focussed on potential intergenerational effects of paternal methyl donor depletion on LR offspring only.

### Paternal dietary methyl donor depletion and generation of first generation offspring

Adult LR male rats were fed standard rat chow (NIH-31 Open Formula 7917 18% protein diet, Harlan Laboratories, Oxon, U.K.) *ad libitum* throughout their lifetime until postnatal day (P)75. At P75, LR males were assigned to one of two diet conditions: (i) control diet (LR-CON); or (ii) chow that was 90% depleted of methyl donors (LR-DEP; *n*=8 LR males per condition). The LR-CON males received commercially made semisynthetic l-amino acid-complete rodent diet number A10021 (Research Diets Inc, New Brunkswick, NJ). The LR-DEP group received an l-amino acid-defined rodent diet lacking 90% of normal requirements of choline, folate, and methionine (diet number A04062402, Research Diets Inc, New Brunkswick, NJ).

After receiving the CON or DEP diet for 5 weeks, LR males were mated with naive LR females that were exposed to standard rat chow (NIH-31 Open Formula 7917 18% protein diet, Harlan Laboratories, Oxon, U.K.) *ad libitum* throughout mating, pregnancy, and the postpartum period. The males were removed from the cage after mating. While maintenance DNA methylation only takes place until meiosis, *de novo* methylation activity occurs through the pachytene spermatocytes phase and DNMT expression studies suggest that this may even occur through the round spermatid phase of spermatogenesis, so our diet treatment should affect much of the spermatogenesis process [32]. When females gave birth (P0), litters were culled to 12 pups (6 males/6 females). Pups that had LR fathers fed the control diet (first generation (F1)-CON) or fathers fed the methyl donor depleted diet (F1-DEP) were weaned on P21 and only males were aged to adulthood for later behavioral assessment (*n*=14 per F1-CON and F1-DEP conditions, with no more than two littermates per groups). The F1 LR male progeny were housed two to three per cage until adulthood (P75) when they embarked on the behavioral test battery described below.

### Behavioral testing

Adult male F1-CON and F1-DEP LR offspring were evaluated in a behavioral test battery comprising several classic rodent tests of anxiety- and depression-related behavior: the open field test (OFT), elevated plus maze (EPM), social interaction test, and forced swim test (FST). Rats were subjected to the full test battery in this test order, with 1–2 days’ rest between tests. Tests were performed between 8 a.m. and 12:30 p.m. and conducted under dim lighting (30 Lux) as previously described [16]. Behavior was recorded with a digital camera, and quantitated in a blinded fashion utilizing Ethovision^®^ XT 8.0 software (Noldus, Wageningen, The Netherlands).

#### OFT

Testing was conducted in a 100 × 100 × 50 cm black Plexiglas box with a black floor. At the beginning of the test, a rat was placed in a corner of the box and was allowed to explore the apparatus for 5 min.

#### EPM

Testing was conducted in a black Plexiglas apparatus with four elevated arms (70 cm from the floor, 45 cm long and 12 cm wide) arranged in a cross. Two opposite arms were open and the other two arms were enclosed by 45-cm high walls. At the start of each test, a rat was placed in the center square of the EPM facing a closed arm, then allowed to freely explore for 5 min.

#### Social interaction

Testing was conducted in a rectangular black Plexiglas box (91 × 61 × 30 cm), which was divided into three chambers separated by dividers with openings in the center to allow animals to move freely between zones. Testing was conducted in over 2 days (10 min/day). On day 1, the test rat was placed in the empty middle chamber and allowed to explore the apparatus for 10 min; one of the other zones contained an empty cylindrical metal bar-cage, and the third zone contained an adult male stimulus rat within an interaction cage placed in a corner. On day 2, the test rat was again placed in the middle chamber and allowed to explore for 10 min; one of the other zones contained a novel male stimulus rat within the interaction cage, and the third zone contained an adult female stimulus rat within its interaction cage. Stimulus rats were age-matched and of the same strain as the test animals.

#### FST

Testing was conducted in clear Plexiglas water chambers (40 cm high × 40 cm diameter) filled with water at 25°C to a depth of 30 cm. On FST day 1, rats were placed (one rat/cylinder) in the water for 15 min (pretest phase); 24 h later the rats were returned to the water-filled cylinder and tested for 5 min (test phase).

### Statistical analysis

Data were analyzed using GraphPad Prism Software (version 6.0 for Windows, GraphPad Software, La Jolla, California, U.S.A., www.graphpad.com). All datasets were first verified to be normally distributed using the D’Agostino and Pearson omnibus normality test. Behavioral data from the OFT, EPM, and FST were analyzed by *t* test to compare F1-CON and F1-DEP LR progeny. If data were not normally distributed, the non-parametric equivalent test was used (for *t* test, Mann–Whitney U test was used). In the social interaction test, we used a two-way ANOVA, with paternal diet condition and social stimulus condition (novel object, male stimulus rat, or female stimulus rat) as independent factors. Tukey’s or Sidak’s multiple comparsions post hoc analyses were used when appropriate. For all tests, α = 0.05.

## Results

Paternal dietary methyl donor depletion did not appear to affect male fertility, since LR-DEP males were able to mate as successfully as LR-CON males (100% pregnancies in mated male/female pairs in both conditions). Likewise, the LR fathers’ diet condition did not affect outcomes for the litters, with no differences in gestational length and no differences in litter size (F1-CON litter size: 13.5 ± 1.37 pups; F1-DEP litter size: 11.9 ± 2.4 pups). The F0 body weights at the time of breeding was lower in the LR-DEP (LR-CON: 486.9 ± 17.06, *n*=16; LR-DEP: 437.2 ± 12.95, *n*=16; t = 2.319, df = 30, *P*=0.0274). The body weight of the F1-DEP was greater before and after behavioral testing was performed (F1-DEP: 362.9 ± 7.675, *n*=14; F1-CON: 337.3 ± 6.168, *n*=14; Interaction: F (1, 26) = 0.01128, *P*=0.05; Time: F (1, 26) = 389.6, *P*≤0.0001; Diet: F (1, 26) = 6.374, *P*=0.018).

**Figure 1:**
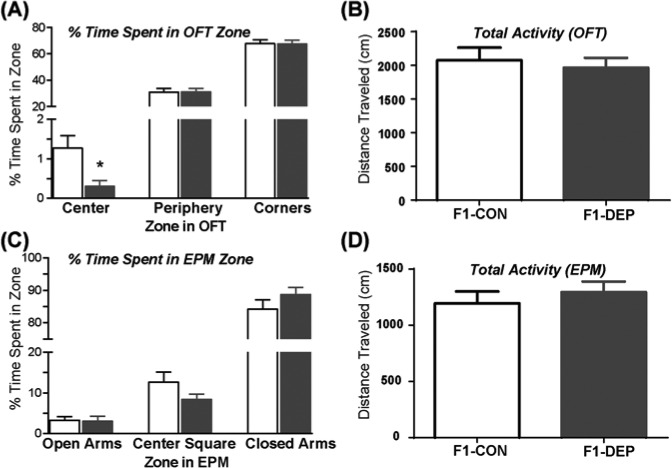
Paternal methyl donor depletion leads to increased anxiety-like behavior in F1 offspring (**A**) In the OFT, progeny of LR fathers that were fed a F1-DEP displayed increased anxiety-like behavior (diminished time in the center of the open field) compared with controls (F1-CON). No differences in time spent in the periphery or corners. (**B**) Paternal diet did not influence total activity in the OFT. (**C**,**D**) In the EPM, paternal methyl donor diet content did not affect time spent in the any zone within EPM or total exploratory activity. Data represent mean ± S.E.M.; * indicates *P*-value <0.05.

When F1-CON and F1-DEP male LR offspring reach adulthood, they were evaluated in an emotional behavioral test battery. In the OFT, LR rats whose fathers had received the F1-DEP showed enhanced anxiety-like behavior, spending less time in the center of the open field relative to controls (F1-CON Mann–Whitney U stat = 41, *P*<0.05). No differences were found in the other zones of the OFT (Figure 1A). Novelty-induced activity in the OFT was similar between the two groups (Figure 1B). Paternal methyl donor diet condition did not affect behavior in the EPM (Figure 1C,D).

**Figure 2:**
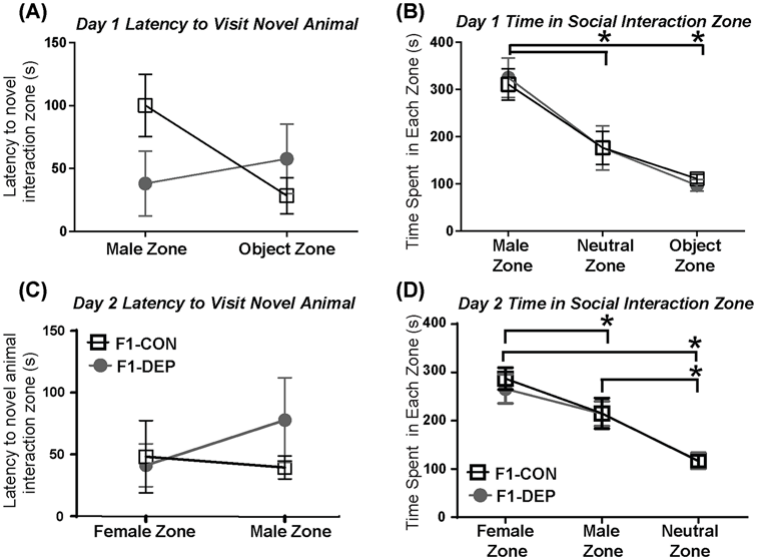
Paternal methyl donor depletion does not affect offspring’s sociability (**A**) On day 1 of social interaction, parental methyl donor depletion did not affect offspring’s latency to visit a novel male stimulus rat or novel object. (**B**) Parental diet did not affect the time spent in close proximity to a novel male rat or object. All rats did, however, spend more time in the novel male zone than the object or neutral zone. (**C**) On day 2 of social interaction, no group differences were found in latency to approach novel female or male stimulus rat. (**D**) Paternal diet did not affect the time spent near the novel female or male rat. All rats preferred to spend time in the female interaction zone over the male and neutral zones and preferred the male interaction zone over neutral zone. Data represent mean ± S.E.M.; * indicates *P*-value <0.05 for post-hoc analysis.

Next, we performed a two-phase social interaction tasks. On the first day, the experimental rats were introduced into the center chamber of a three-chamber arena with a novel male in a separate chamber and a novel object (empty cage) in the opposite side chamber. There were no group differences in the latency to approach the novel male or object (Figure 2A) or in the duration of the interaction (Figure 2B). There was a main effect of duration in zone visited (two-way ANOVA: F(2, 78) = 21.60, *P*<0.0001). Through post-hoc analysis, both experimental groups were found to prefer the novel male over both the object zone and the neutral (center) zone (Tukey’s: *P*<0.05 for time spent exploring male compared with object or neutral zone) was found. On the second day, a novel male was in a side chamber and a novel female was in the opposite chamber. There were no group differences in latency to visit the male or female stimulus rat (Figure 2C). When we considered time spent in close proximity to either the female stimulus rat, male stimulus rat, or empty chamber in the social interaction chamber, both experimental groups showed preference for the female stimulus rat (Figure 2D; effect of social stimulus, F(2, 77) = 22.28; *P*<0.001; post-hoc analysis *P*<0.05 for time spent exploring female compared with male or neutral zone and male zone compared with neutral zone).

**Figure 3:**
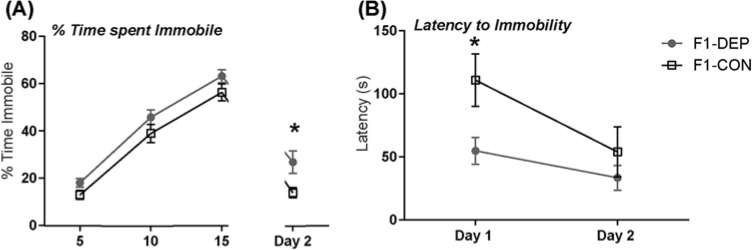
Paternal methyl donor depletion increases helpless behavior (**A**) In the FST, offspring from LR fathers fed the F1-DEP showed significantly higher immobility duration in the FST (an indicator of behavioral despair and depression-like behavior) compared with controls (F1-CON) during day 2 testing period. Main effects of day and treatment were found through two-way ANOVA. (**B**) In FST, the latency to first exhibition of immobility was decreased in F1-DEP compared with F1-CON on day 1. Main effects of day and treatment were found in two-way ANOVA. Data represent mean ± S.E.M.; * indicates *P*-value <0.05 for post-hoc analysis.

Finally, in the FST, paternal methyl donor dietary depletion led to increased depression-like behavior (immobility), with F1-DEP LR offspring showing greater immobility compared with F1-CON LRs through the first and second day of testing (Figure 3A; 2-way ANOVA, Time: F(3, 104) = 84.84, *P*<0.0001; *Treatment*: F(1, 104) = 12.74, *P*<0.001)). Through post-hoc analysis, F1-DEP showed a greater immobility duration in day 2 testing phase (Figure 3A; Sidak’s, t(104) = 2.906, *P*<0.05). Through latency to immobility, an effect was found in day and treatment (two-way ANOVA, Day: F(1, 26) = 6.130, *P*<0.05; Treatment: F(1,26) = 5.561, *P*<0.05). F1-DEP were found to have a lower latency to immobility in day 1 of FST (Figure 3B; Sidak’s, t(52) = 2.475, *P*<0.05).

## Discussion

Our previous work identified DNA methylation differences in the brains of rats bred to display disparate emotional behavior [16,30,31], and we found that dietary methyl donor depletion leads to exaggerated anxiety- and depression-like behavior in adult male rats already prone to a high anxiety/depression-like phenotype [16]. The present study examined whether dietary methyl donor depletion in male rats prior to mating would trigger intergenerational effects on emotional behavior in F1 offspring. We hypothesized, and indeed found, that F1 male offspring of methyl donor depleted fathers (F1-DEP) showed a similar behavioral profile to the F0 males, including increased anxiety-like behavior in the OFT and enhanced depression-like behavior (immobility) in the FST. Paternal methyl donor deficiency did not affect F1 offspring’s novelty-induced activity, behavior in EPM or social behavior in our social interaction paradigm. Due to the small yet significant change in the OFT and no observed differences in the EPM, it may be that the paternal diet is only contributing slightly to the anxiety-like behaviors and is not able to alter the behaviors in the more stressful circumstances that may be more difficult to overcome as such in EPM. As LR rats typically show high levels of inhibited behaviors in such tasks, there is a possibility of a floor effect leading to an inability to find difference between inhibited phenotype in a stress-evoking task as EPM. In our previous study that included the F0 males, we tested the effects of both methyl donor depleted and supplemented diets. Here, we found that methyl donor supplementation increased time spent in the open arms of the EPM (showing less anxiety-like behavior). In FST, we found that DEP lead to increased time spent immobile (increased depression-like behavior) and a non-significant trend for supplementation to lead to improvement in this task [16].

There is a growing body of evidence demonstrating how environmental factors, including nutrition as well as exposure to stress and environmental toxins, shape paternal influences on offspring [33,34]. There is limited information to-date regarding the effects of paternal dietary methyl donor content on offspring, however. A recent study showed that paternal methyl donor supplementation increased DNA methylation levels in offspring’s brains, disrupted hippocampal synaptic plasticity and triggered learning and memory deficiencies [25]. Another study evaluated the effects of paternal methyl donor depletion; it found that paternal methyl donor depletion lead to decreased DNA methylation in offspring’s liver and brain, but did not assess behavioral consequences [24]. Thus, our study represents an important contribution to the literature, as it is the first to demonstrate intergenerational behavioral effects in F1 offspring of fathers exposed to dietary methyl donor depletion. Future studies will explore molecular changes in the brains of F1 offspring to delve into mechanisms that drive the observed changes in anxiety- and depression-like behavior.

An important limitation of the present study is that we focussed only on male F1 offspring. In general, the literature lacks information regarding epigenetic (including DNA methylation) differences in the brains of males compared with females [35,36] and how such differences may contribute to sexually dimorphic risk for emotional dysfunction [37]. Thus, it will be important for future studies to examine intergenerational effects of paternal dietary methyl donor depletion in both male and female offspring. Our previous work with the HR/LR model shows that LR females display high levels of anxiety- and depression-like behaviors akin to their male LR counterparts [26,38,39]. We therefore predict that future studies in LR females will identify DNA methylation changes that contribute to their behavioral phenotypes, and that their brain and behavior could be modified by dietary and other manipulations known to influence neural DNA methylation levels. Another caveat of the present study is that we only examined the effect of paternal dietary methyl donor depletion on F1 LR offspring. We chose to focus on LRs because our prior work showed that manipulating DNA methylation in LRs successfully altered their behavior [16]. It would be interesting to repeat our study in HR rats to determine whether they would be similarly affected by the manipulation. Alternatively, it could also be useful to repeat the study in a group of ‘normal’ outbred Sprague–Dawley rats, which are known to display an intermediate behavioral phenotype relative to the selectively bred HR/LR lines [40].

## Conclusion

In summary, our previous work identified numerous gene expression and epigenetic differences (including altered DNA methylation) in the brains of rats that display disparate emotional behavior phenotypes [16,27–30]. We later found that changing DNA methylation via manipulation of dietary methyl donor content could improve or worsen anxiety- and depression-like behavior in LR rats that typically display a high depression/anxiety-like phenotype [16]. We now show that this dietary manipulation (methyl donor depletion) evokes intergenerational effects, with F1-DEP LR offspring showing a similar worsening of their anxiety- and depression-like behavior relative to controls. Ongoing studies will examine molecular and neural changes in the brains of paternal dietary methyl donor depleted F1 offspring to better understand intergenerational epigenetic mechanisms that shape brain function and behavior.

## Abbreviations

CON: control
DEP: methyl donor depleted diet
DNMT: DNA methyltransferase
EPM: elevated plus maze
F1: first generation
F0: parental generation
FST: forced swim test
HR: high novelty responder rat
LR: low novelty responder rat
OFT: open field test
SAM: *S*-adenosylmethionine
